# Perceptions of vaccine preventable diseases in Australian healthcare: focus on pertussis

**DOI:** 10.1080/21645515.2020.1780848

**Published:** 2020-07-22

**Authors:** Julianne Bayliss, Roshnee Randhawa, Kyu-Bin Oh, Walid Kandeil, Victoria A. Jenkins, Elisa Turriani, Michael Nissen

**Affiliations:** a Medical Affairs, GSK, Melbourne, Australia; b Medical Affairs, GSK, Singapore, Singapore; c Global Medical Affairs, GSK, Wavre, Belgium

**Keywords:** Pertussis, whooping cough, vaccination, adults, Australia, recommendations, general practitioners, survey

## Abstract

Adult vaccination in Australia is suboptimal. For instance, as few as one in nine people have received a pertussis vaccine in adolescence or adulthood, despite increasing disease burden and evidence of a positive correlation between older age and hospitalization rates. The objectives of this study were to describe general practitioners’ (GPs) and adult consumers’ knowledge and attitudes toward adult vaccination, with an emphasis on pertussis. Australian GPs and consumers were recruited in two nationally representative online surveys repeated annually between 2014 and 2018. Vaccination discussions occurred in a minority of adult/GP encounters. Pertussis was among the five most frequently identified vaccine preventable diseases but was unlikely to be proactively discussed with adults not in contact with young children. Among consumers, only one in three recalled ever receiving a pertussis vaccination. GPs are a strong predictor of adults receiving a pertussis vaccine. Possible factors contributing to low uptake are misconceptions around pertussis disease, vaccination requirements and lack of GP recommendation for adult vaccination. GPs have a key role to play in increasing adult vaccination coverage with their recommendation.

## Introduction

Immunization coverage rates in Australian children are among the highest in the world, with over 90% of children receiving all recommended vaccines by the relevant age milestones.^1-3^ A number of factors contribute to this high uptake, including the comprehensive funding of the childhood immunization schedule by the government through the National Immunization Program (NIP),^4^ the Australian Childhood Immunization Register (recently expanded to all ages and renamed), which has tracked the vaccination status of children at an individual level since 1996;^1^ and an adapted legislation preventing the collection of government family benefits and enrollment of children into childcare facilities without proof of immunization.^3^

The national recommendations for vaccination are made by the Australian Technical Advisory Group on Immunization (ATAGI) and listed in the Australian Immunization Handbook (AIH).^5^ They include funded and nonfunded vaccines. For defined adult groups, the NIP provides influenza (annual), pneumococcal, zoster and diphtheria-tetanus-pertussis vaccinations.^4^

Regardless of whether the vaccines are funded through the NIP or privately purchased, adult coverage remains low for several vaccines and target populations.^3,6^ For instance, as few as one in nine people have received a pertussis vaccine in adolescence or adulthood.^6^

Pertussis is highly contagious in unvaccinated individuals and periodic booster doses are recommended as neither natural nor vaccine-induced immunity against pertussis is lifelong.^5,7^ Over the last two decades, a growing proportion of pertussis notifications have occurred in Australian adults.^8^ There is a correlation showing older age is associated with an increase in hospitalization rates due to complications arising following pertussis infection.^9,10^ ATAGI recommends vaccination to any adult who wishes to reduce their likelihood of becoming ill with pertussis. In addition to this, there are recommendations for vaccination of specific risk groups, either because they are at increased risk of complications themselves, or at increased risk of transmitting pertussis to others who are at the greatest risk of severe complications. This includes pregnant women, older adults (≥50y and ≥65y), travelers, and any persons susceptible to transmit pertussis to vulnerable populations: healthcare workers, early childhood educators, and adult household contacts of children.^5^ However, only maternal immunization is included on the NIP.^4^

We conducted two types of nationally representative surveys in Australia: one of vaccinating general practitioners (GPs) and the other with consumers. The GPs were questioned about their vaccinating behavior, especially against pertussis. The consumers were asked about their perception of a GP’s role in recommending vaccination and factors influencing their uptake of vaccines with a special focus on pertussis, including their knowledge about risk of contracting the disease. Our overarching goal is to understand how pertussis vaccine uptake can be improved in Australian adults, particularly in those at increased risk of complications.

## Material and methods

### Sampling and data collection

A market research survey of Australian GPs and consumers about vaccine preventable diseases (VPDs) was conducted by the company IQVIA, who were contracted by GSK. Surveys were conducted four times to provide representative samples of behaviors across a four-year time frame. For GPs, the individual surveys were completed in June 2015 (wave 1), April 2016 (wave 2), May 2017 (wave 3), and September 2018 (wave 4). For consumers, the individual surveys were conducted in September 2014 (wave 1), October 2015 (wave 2), July 2016 (wave 3), and September 2018 (wave 4).

All participants provided informed consent to use of the results of the market research in an external context at an aggregated level in accordance with the Medicines Australia Code of Conduct and the Australian Market and Social Research Code of Professional Behavior. As the study did not involve any medical intervention on human or animal subjects, an approval by an ethics committee was not requested.

#### GP survey

For each survey wave, a nationally representative sample of approximately 100 Australian GPs was drawn randomly from a field force list and from a fieldwork panel provider’s sample (Supplementary Table S1). GPs were compensated in line with local fair market value payment as per the Medicines Australia Code of Conduct.^11^

GPs were eligible for survey wave 1 if they provided vaccination advice to adult patients at least once a month. The eligibility criterion was further refined for waves 2–4 such that a GP had to see at least 400 patients per month, provide vaccination advice (excluding travel vaccinations) to adult patients at least weekly and to at least 5 patients per month.

#### Consumer survey

The consumers were recruited via a third-party panel provider. The provider instituted panel recruitment and maintenance policies to ensure the panel population was representative of the Australian population on demographic and behavioral variables. Invitations were sent randomly to people on the panel and screening questions applied to ensure participants belonged to any of the following groups (Supplementary Table S1). These are groups subject to specific vaccine recommendations in Australia:^5^

- People aged 50–64 years
- People aged 65 years and over, excluding those in close contact with children aged less than 5 years
- Grandfathers of children under 5 years
- Grandmothers of children under 5 years
- Fathers of children under 5 years
- Mothers of children under 5 years
- People traveling overseas in the next 6 months
- High risk workers (which include childcare workers and tetanus-prone workers, i.e. construction/agriculture workers)

Consumers who completely disagreed with the statement “Vaccinations are important and help control disease” were excluded from completion of the entire survey.

### Statistical analyses

Statistical analyses were performed using Stata 13.1 for Windows. Normally distributed data was reported as mean ± standard error. Non-parametric data was reported as median (range). Categorical data was reported as number (percentage). Univariate analysis of outcome variables was conducted using t-tests, Wilcoxon Rank Sum and Fisher’s exact test as appropriate. Univariate comparisons between multiple patient groups were undertaken using analysis of variance (ANOVA) or Kruskal-Wallis tests as appropriate. In all cases a *p*-value of 0.05 was considered statistically significant.

## Results

a total of 412 GPs and 6529 adult consumers were interviewed across the four survey waves (Supplementary Table S1). More than 80% of GPs interviewed obtained their medical degree before 2000 and more than 50% belonged to middle- (4–8 GPs) and large- (> 8 GPs) sized medical practices. Consumers were assessed as belonging to one of eight separate patient segments. For both GPs and consumers, the geographical distribution of respondents was similar to that of the general population^12^ and did not vary significantly between study waves.

### GP attitudes toward adult vaccination

**Figure 1.**
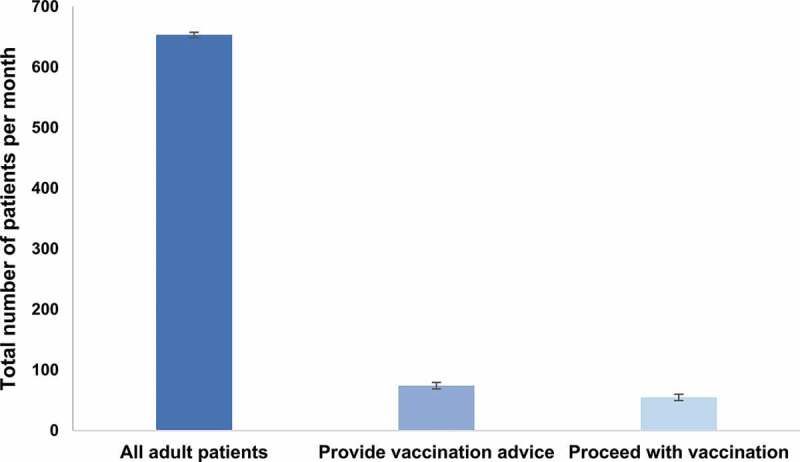
Average monthly number of adult patients in consultation at their general practitioners, receiving vaccination advice and proceeding with vaccination, 2015–2018

Across survey waves 1–4, GPs indicated discussing vaccination with 9–13% of their adult patients. When vaccination was discussed, GPs indicated that 69–77% of patients will proceed to be vaccinated according to their recommendations. Based on these estimations, 6-10% of the total population of adult patients visiting their GP would be vaccinated each year (Figure 1).

Footnote: Data is reported as mean of the means for each survey wave ± standard error of the mean.

**Figure 2.**
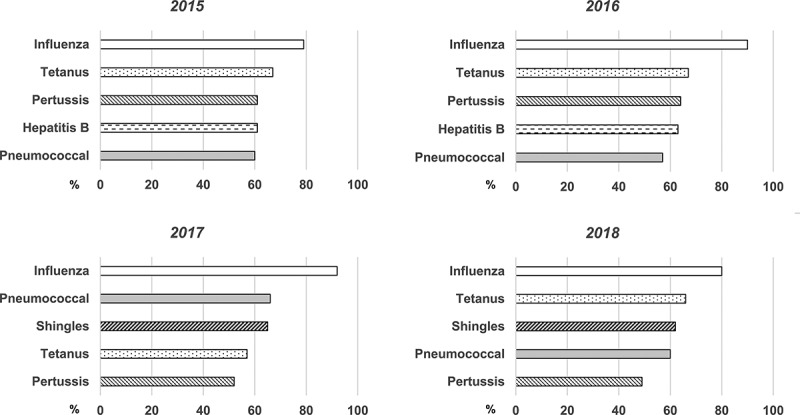
Five most frequently identified vaccine preventable diseases discussed by general practitioners with their adult patients, 2015–2018

Throughout the study period, influenza remained the most frequently discussed VPD with the median proportion of GPs who spontaneously recalled discussing influenza vaccination being 85% (range 79-92%) across waves 1–4. In contrast, pertussis, while still within the top five recalled VPDs, was only spontaneously identified by a median of 56.5% of GP respondents (range 49-64%) across waves 1–4 (Figure 2).

Footnote: Data is reported as median proportion of general practitioners who spontaneously recalled discussing specific vaccinations. Pneumococcal: pneumococcal disease vaccination.

Across all four study waves, GPs identified themselves as the primary initiators of pertussis vaccination discussions in general practice, taking the initiative in 52-57% of encounters. When asked about patient types to be targeted for pertussis vaccination, GP respondents were more likely to identify individuals in close contact with children <5 years of age (parents, grandparents and family members) as needing pertussis vaccination, compared to those not in close contact with children <5 years of age (older adults and travelers), or in the setting of illness/outbreak. Further, GPs were twice as likely to recommend pertussis vaccination for grandparents compared to adults of a similar age who were not in regular contact with children (*p* < 0.0001). Discussion of pertussis vaccination in the setting of disease outbreak or underlying patient illness remained uncommon.

### Consumer attitudes and knowledge toward adult vaccination

The majority of survey respondents demonstrated a positive attitude toward vaccination, with >90% of respondents indicating they strongly or completely agreed with the statement that “Vaccinations are important and help control disease”. Less than 5% of respondents disagreed with the importance of vaccination. These individuals were excluded from further questions.

When questioned as to where they would go for information on vaccination, consumers consistently cited GPs as the most reliable resource; identified by >86% of respondents across all four study waves. This included those individuals who reported never having received a vaccine as an adult. Unvaccinated adults identified a lack of GP recommendation (52%) and lack of awareness of the need for adult vaccination (26%) as the two top reasons for not having any vaccinations as an adult.

**Figure 3.**
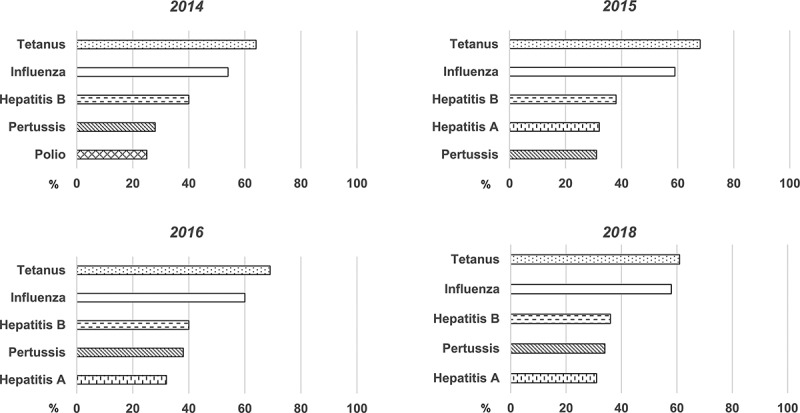
Five most frequently recalled adult vaccinations recalled by consumers, 2014–2018

Among those adults who recalled being vaccinated at any time during adulthood, the five most frequently identified vaccines were tetanus, influenza, hepatitis B, hepatitis a and pertussis (Figure 3). Overall there was little change in the vaccines identified over subsequent waves of the study. Tetanus vaccination was the most frequently identified vaccine received at any time as an adult and was more than twice as likely to be recalled than pertussis vaccination (66% (61–69%) vs. 33% (28–38%) respectively; *p* < 0.0001). Likewise, significantly more respondents reported ever having received an influenza vaccination as an adult, compared to pertussis vaccination (59% (54- 60%) for influenza; *p* < 0.0001).

Footnote: Data is reported as median proportion of consumers who recalled specific vaccination. Polio: poliomyelitis vaccination.

**Figure 4.**
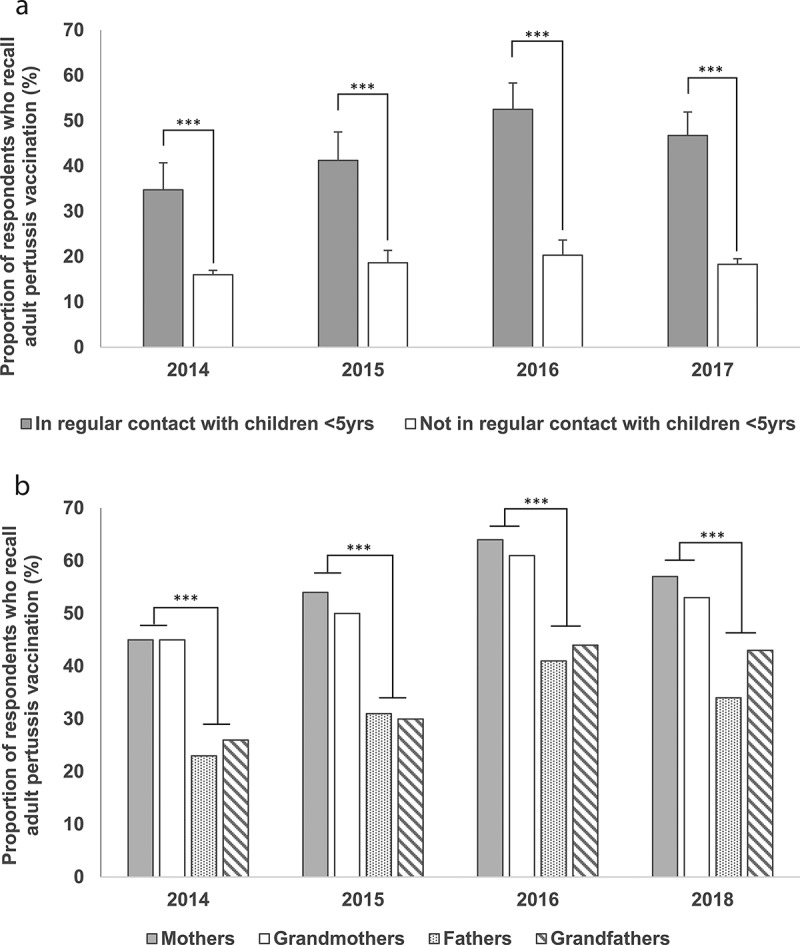
(a and b) Proportion of survey respondents who recall pertussis vaccination, per population segments, 2014–2018

Individuals in close contact with children <5 years old (parents, grandparents and carers of children) continued to show the highest recall rates of adult pertussis vaccination over time (Figure 4(a)). In contrast, those adult populations without regular contact with young children (50–64 years, 65 years and over and travelers) were significantly less likely to recall ever having received a pertussis vaccination as an adult (*p* < 0.0001 for all) and recall rates did not change significantly over time. Of those respondents who were in close contact with children, mothers and grandmothers consistently reported significantly higher rates of pertussis vaccination than fathers and grandfathers (*p* = 0.001; Figure 4(b)).

Footnote: Data is reported as mean of the means for each survey wave ± standard error of the mean. Individuals in regular contact with children <5 yrs (under 5 years old): parents, grandparents and carers of children. Not in regular contact with <5 yrs: 50–64 years, 65 years and over and travelers. *** *p*-value < 0.001.

## Discussion

This study explored the perceptions and attitudes toward adult vaccination of Australian GPs and vaccine consumers through two country-wide surveys. The surveys were repeated four times between 2014 and 2018 to allow for the assessment of changes in perceptions and behaviors over time. a special focus was given to adult pertussis vaccination.

Our results highlight that discussions around adult vaccination only occur in a minority (<10%) of GP encounters, with suboptimal involvement of GPs in adult pertussis immunization management. Adult immunization was most commonly associated with influenza and tetanus, as well as hepatitis Aand B and pertussis to a lesser extent. Further, there was no observable change in this behavior over time.

Booster vaccination of adults aged ≥65 years against pertussis is a specific recommendation of the AIH.^5^ This recommendation is based on documented evidence supporting more severe pertussis morbidity associated with age^9^ and with more frequent pertussis-related complications in adults than in adolescents (in 28% and 16% of pertussis cases, respectively).^13^ Specifically, an Australian study described significant association of age and pertussis-related hospitalization and calculated that 4.0–5.2% of patients aged ≥45 years and up to 10% of adults aged ≥65 years with a pertussis diagnosis were hospitalized during a large outbreak between 2006 and 2008.^10^

GPs’ higher likelihood to proactively check the pertussis vaccination status of their older patients with grandchildren compared to those without remained consistent throughout the study. GPs were twice as likely to recommend pertussis vaccination for grandparents as for adults of a similar age without grandchildren. Additionally, over the course of the study, the number of pertussis-related discussions fell from the third to the fifth most commonly discussed vaccine preventable disease in adults (61% in 2015 to 49% in 2018). This change may also be related to the AIH recommendation for maternal immunization over cocooning for pertussis issued in March 2015 and the progressive replacement of cocooning programs in subsequent years, as well as the introduction of a funded zoster vaccination program from November 2016.^14,15^

In contrast to GPs’ declining tendency to check pertussis vaccination status in adults >50 years over the study period, a growing proportion of grandparents reported pertussis vaccine uptake during the same time frame. Thus, despite the changes in GPs’ behavior, people who are in close contact with young children continue to abide by the immunization recommendations. This is a possible result of a rise in proactive requests for vaccination by these patients or access to vaccination through other immunization providers such as nurses and pharmacists. In addition, there are significantly more grandparents to recall pertussis vaccination in adulthood than older adults without grandchildren. Among the latter, barely one in five expressed moderate to strong concern about the impact of pertussis disease to themselves. Taken together, these findings suggest that pertussis continues to be considered as a disease of childhood and infancy, with consumers and healthcare professionals alike, failing to recognize the risks associated with pertussis infection as an adult. There is evidence that suggests pertussis is underdiagnosed,^5,16,17^ among 25% of adults who experienced a cough lasting at least 5 days, up to 7% may be caused by *Bordetella pertussis*.^5^ This emphasizes the key role of GPs in optimizing adult vaccination uptake through their recommendations.^3^

Our results identified GPs as the single most trusted source for vaccination advice amongst Australian adults. This is evidenced through both the willingness of individuals to receive vaccination advice from their GP, and through the GP’s own recollection that on average at least 75% of patients with whom immunization is discussed will proceed to be vaccinated. Other studies have likewise demonstrated that the most important factor influencing vaccination uptake in older patients is a recommendation from a healthcare professional.^3^

It is important to recognize the limitations of the current study. Firstly, consumers who identified as being anti-vaccination were excluded from the completion of the entire survey. Secondly, some survey questions included a list of predefined answers, which could bias the responses. Finally, recall bias is inherent to this type of study; for instance, vaccination rates were self-reported and not retrieved from any health clinic/pharmacy register or personal vaccination record, and therefore should be considered with caution. Nevertheless, this approach fits the study purpose of assessing respondents’ perceptions and attitudes toward vaccination.

Taken together, our data suggest a continued misperception that pertussis is a childhood disease, combined with a general lack of adult awareness of increased risk of pertussis and disease severity, resulting from a lack of vaccination recommendation from GPs.

These barriers could be overcome by engaging adult patients in discussions around the increased risk of infection by VPDs and severe outcomes with aging, especially as there exists a common misconception that a healthy lifestyle is sufficient to prevent VPDs in the elderly.^18^ Several factors are significantly associated with adults’ likelihood to be vaccinated, including a positive perception of vaccination, greater perception of disease severity, self-perception of high susceptibility and recommendation from the healthcare provider.^19-21^

## Conclusions

**Figure 5.**
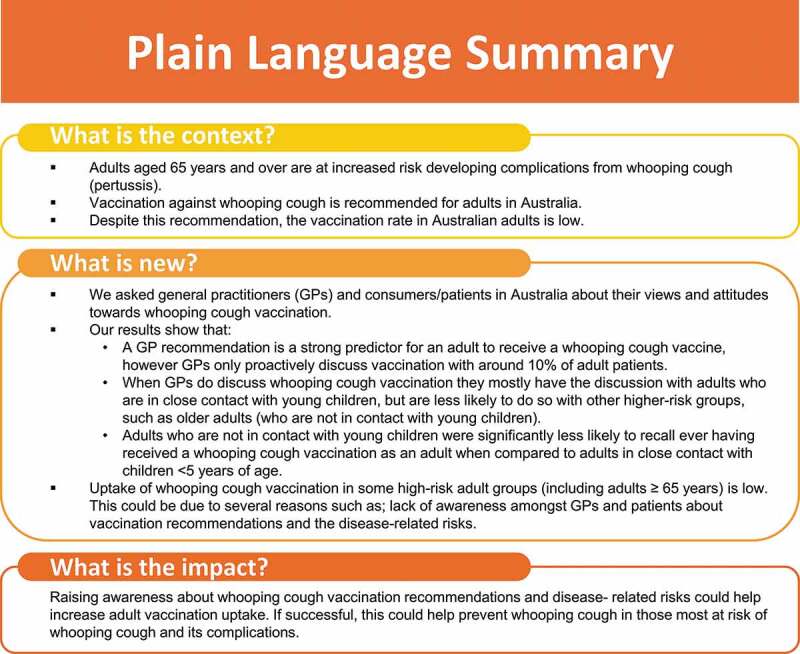
Plain Language Summary

Although a large majority of Australian adults declare being in favor of vaccination, only one in three survey respondents recalled ever receiving a pertussis vaccine in adulthood. Pertussis vaccination was most likely to be recommended by GPs for adults in close contact with infants. Pertussis seemed misconceived as a childhood disease and its associated risks in adults disregarded. Regular vaccination discussions, review of adult patients’ vaccination status and formulation of recommendations should improve patient knowledge of adult vaccination and awareness of risk, and increase vaccine uptake in adults.^18,21^ Figure 5 provides a plain language summary of the findings of this study.

## Supplementary Material

Supplemental Material
