# Supplementary material for: Perceptions of vaccine preventable diseases in Australian healthcare: focus on pertussis
Source: Hum Vaccin Immunother. 2020 Jul 22;17(2):344–50. doi: 10.1080/21645515.2020.1780848 (PMC7899657; doi:10.1080/21645515.2020.1780848)
Supplement: Supplemental Material [file KHVI_A_1780848_SM5849.docx]

**Supplementary Table S1.** Respondent characteristics in the GP survey (A) and in the consumer survey (B)

| **A. General practitioners (GPs)** | **Summary statistics, n (%)** | | | |
| --- | --- | --- | --- | --- |
|  | **2015, n= 100**  **Wave 1** | **2016, n= 106**  **Wave 2** | **2017, n= 105**  **Wave 3** | **2018, n= 101**  **Wave 4** |
| **Gender** |  |  |  |  |
| Male | 71 (71) | 76 (72) | 75 (71) | 65 (64) |
| Female | 29 (29) | 30 (28) | 30 (29) | 36 (36) |
| **Location of practice** |  |  |  |  |
| ACT | 1 (1) | 2 (2) | 3 (3) | 0 |
| NSW | 38 (38) | 34 (32) | 41 (39) | 34 (34) |
| NT | 1 (1) | 0 | 0 | 1 (1) |
| QLD | 22 (22) | 22 (21) | 14 (13) | 24 (24) |
| SA | 8 (8) | 5 (5) | 10 (10) | 10 (10) |
| TAS | 0 | 3 (3) | 2 (2) | 4 (4) |
| VIC | 28 (28) | 34 (32) | 27 (26) | 20 (20) |
| WA | 2 (2) | 6 (6) | 8 (8) | 8 (8) |
| **Year medical degree was obtained** |  |  |  |  |
| 2000 onwards | 17 (17) | 22 (21) | 15 (14) | 20 (20) |
| 1990-1999 | 24 (24) | 28 (26) | 36 (34) | 30 (30) |
| 1980-1989 | 40 (40) | 35 (33) | 35 (33) | 29 (29) |
| 1970-1979 | 18 (18) | 20 (19) | 16 (15) | 17 (17) |
| Before 1970 | 1 (1) | 1 (1) | 2 (2) | 4 (4) |
| Prefer not to answer | 0 (0) | 0 | 1 (1) | 1 (1) |
| **Practice size** |  |  |  |  |
| 1 GP | 16 (16) | 9 (8) | 11 (10) | 8 (8) |
| 2-3 GPs | 27 (27) | 13 (12) | 12 (11) | 15 (15) |
| 4-8 GPs | 31 (31) | 42 (40) | 33 (31) | 46 (46) |
| > 8 GPs | 23 (23) | 33 (31) | 42 (40) | 27 (27) |
| GP super clinic/corporate clinic | 3 (3) | 9 (8) | 7 (7) | 5 (5) |
|  | |  |  |  |
| **B. Consumers** | **Summary statistics, n (%)** | | | |
|  | **2014, n= 1538**  **Wave 1** | **2015, n= 1726**  **Wave 2** | **2016, n= 1523**  **Wave 3** | **2018, n= 1540**  **Wave 4** |
| **State of residence** |  |  |  |  |
| ACT | 49 (3) | 35 (2) | 61 (4) | 30 (2) |
| NSW | 514 (34) | 535 (31) | 472 (31) | 477 (31) |
| NT | 5 (0) | 0 | 15 (1) | 2 (<1) |
| QLD | 352 (23) | 362 (21) | 289 (19) | 308 (20) |
| SA | 121 (8) | 138 (8) | 137 (9) | 123 (8) |
| TAS | 47 (3) | 52 (3) | 61 (4) | 46 (3) |
| VIC | 325 (22) | 449 (26) | 381 (25) | 400 (26) |
| WA | 125 (8) | 155 (9) | 107 (7) | 154 (10) |
| **Consumer segment*** |  |  |  |  |
| Mothers | 203 (13) | 234 (14) | 205 (13) | 208 (14) |
| Fathers | 205 (13) | 223 (13) | 201 (13) | 202 (13) |
| Grandmothers | 205 (13) | 231 (13) | 202 (13) | 210 (14) |
| Grandfathers | 205 (13) | 227 (13) | 202 (13) | 209 (14) |
| 50-64 | 206 (13) | 240 (14) | 202 (13) | 203 (13) |
| ≥65 | 205 (13) | 233 (13) | 202 (13) | 201 (13) |
| Travelers | 204 (13) | 232 (13) | 207 (14) | 202 (13) |
| High risk workers^ | 105 (7) | 106 (6) | 102 (7) | 105 (7) |

ACT: Australian Capital Territory; GP: General Practitioner; NSW: New South Wales; NT: Northern Territory; QLD: Queensland; SA: South Australia; TAS: Tasmania; VIC: Victoria; WA: Western Australia

*In 2014 an additional consumer segment of “women planning pregnancy” (n = 202) was included as local immunization practice supported cocooning strategies to protect against pertussis. This segment was removed in 2015 following the implementation of maternal immunization programs.

^High risk workers include childcare workers (high risk of pertussis transmission) and tetanus prone workers.

Percentages might not add up to 100% due to rounding.
